# Commentary: The future of U.S. social prescribing: foundations, implementation, and leadership summit insights

**DOI:** 10.3389/fpsyt.2026.1913990

**Published:** 2026-08-10

**Authors:** Jared Kyle Sieusahai

**Affiliations:** Faculty of Medicine and Dentistry, University of Alberta, Edmonton, AB, Canada

**Keywords:** arts prescribing, community-based intervention, help-seeking, male mental health, masculinity norms, men’s sheds, music therapy, social prescribing

Siegel, Zuckerman, Hashmi, and Shapard’s perspective on the future of social prescribing (SP) in the United States identifies a convergence of crises, an aging population, rising mental illness, and epidemic loneliness, that current clinical infrastructure cannot adequately address (1). Their synthesis makes a compelling case for SP as a low-risk, high-value adjunct to clinical care that can connect patients to community-based social, cultural, and physical activity programs. The paper correctly identifies arts prescribing as an area with significant momentum and research foundations, and it calls for alignment with health equity priorities and attention to vulnerable populations including older adults and those with mental health challenges. This commentary extends their argument in a direction that the paper does not explicitly address: the specific challenge of reaching men through social prescribing, and the evidence that masculinity-congruent community programs represent the most promising and underutilized strategy for doing so.

Men experience substantially elevated rates of undertreated depression, anxiety, social isolation, and suicide relative to women, yet consistently underutilize formal mental health services across the life course (2, 3). The epidemiological burden that Siegel et al. (1) document, including the 23.2% of U.S. adults with mental illness of whom only half receive any treatment, and the epidemic of loneliness affecting one-third of U.S. adults, is disproportionately borne by men in ways that conventional SP frameworks do not adequately account for. It is worth noting that this gap in the SP literature reflects an omission rather than opposition on the part of SP advocates: Siegel et al. (1) do not explicitly exclude men but focus on broad implementation priorities. This commentary is offered as a constructive extension of their framework, not a critique of it. The barriers are not primarily logistical. They are normative: traditional masculinity norms emphasizing self-reliance, emotional stoicism, and strength create a framework within which acknowledging psychological distress and accepting referral to a community support program are experienced as incompatible with masculine identity (4, 5). Masculinity scholars rightly note that there is no single masculine identity; masculine norms vary substantially by culture, class, age, ethnicity, and individual disposition (e.g., Men and Masculinities journal). This plurality is clinically relevant: masculinity-congruent SP programs must be sensitive to the specific masculine norms operating in their target populations rather than assuming a uniform model of manhood. A link worker who assesses a man’s social isolation and refers him to a community connection program faces not only the awareness and stigma barriers that Siegel et al. (1) identify for all patients, but an additional layer of gender-specific resistance that will not yield to better referral pathways or digital platforms alone.

The solution is not to refer men to generic social prescribing programs but to develop and fund masculinity-congruent community programs that reach men through activities they already value, in social contexts that do not require them to identify as help-seekers. As founder of Edmonton Men’s Shed, one of the first Men’s Sheds established in Alberta, the author writes from direct experience of this dynamic: the majority of Shed members who joined in the program’s first year had not previously sought any formal mental health or social support, and the Shed’s practical, project-based framing was the explicit reason they felt comfortable attending. The Men’s Sheds movement, which has grown from Australia across Ireland, Scotland, the United Kingdom, Canada, and beyond to encompass thousands of community-based organizations where men engage in shared practical activities including woodworking, metalwork, and electronics, provides the most extensively documented model of this approach (6, 7). A mixed-methods systematic review of 52 Men’s Shed studies concluded that Shed participation is consistently associated with reduced social isolation and improved subjective wellbeing, particularly for men aged 50 and over (8). It is important to acknowledge that the Men’s Sheds evidence base is heavily concentrated in this older demographic; the evidence for younger men and for men not well served by a work-analog, project-based model is substantially thinner and represents an important gap for future research and program development. A path-model survey of 333 Shed members demonstrated that psychological safety within the Shed predicts engagement, engagement predicts expanded social networks and meaning in life, and both predict improved wellbeing and lower loneliness (9). Canadian evidence from rural Alberta specifically documents that Sheds support older men’s mental health through work-like, project-based activity that provides the camaraderie, purpose, and social inclusion that retirement disrupts, while costing a fraction of formal mental health services (10).

The mechanism that makes Men’s Sheds effective for social prescribing is precisely what Siegel et al. (1) identify as the core SP value proposition: connecting people to community-based activities that address the social drivers of health without requiring clinical framing. What the broader SP literature has not yet articulated clearly is that for men, the specific character of the community activity matters: it must be masculinity-congruent. An activity-based, practical, productive, and group-oriented program that does not foreground health, wellness, or emotional support as its purpose will engage men who decline referrals to social connection groups, mindfulness programs, or community mental health supports framed in clinical terms. This is not a resistance to be overcome through better communication but a structural feature of how diverse masculine identities interact with help-seeking that program design must accommodate rather than ignore.

The same principle applies to arts prescribing, which Siegel et al. (1) identify as a particularly well-evidenced SP modality. The arts prescribing literature has developed primarily around activities with lower masculine valence, including visual arts, dance, and creative writing; to the author’s observation, this literature has more often studied female or mixed-gender populations than male-specific ones, though a comprehensive gender audit of arts prescribing participant demographics has not been conducted. Music therapy and community music programs represent an important exception: observational evidence and clinical reports suggest that men engage with music-based programs at higher rates when the musical format aligns with masculine cultural preferences, though systematic data on gendered engagement rates in arts prescribing remain limited (11, 12). A programmatic evaluation of a music therapist-led community program for veterans in supported housing found that participants strongly preferred rock, country, blues, and folk music, and most frequently engaged through singing and instrument play, active, skill-based, productive modalities that fit within a masculine-coded framework of doing rather than receiving (12). A randomised controlled trial testing the effects of a music video specifically designed to encourage men to seek help found that help-seeking intentions improved in both arms, with no significant between-group difference, indicating a null result on the primary outcome (13). However, the broader evidence on masculinity-congruent media-based interventions is more encouraging: King et al.’s (16) RCT of the Man Up documentary, a three-part series exploring masculinity and suicide, found significant improvements in help-seeking intentions among men who viewed it relative to controls, as well as a significant reduction in adherence to traditional masculine norms. Taken together, this literature suggests that media-based interventions can shift masculine norms, but that the specific format and framing matter considerably.

Siegel et al. (1) identify several critical barriers to SP implementation including limited awareness, fragmented referral pathways, measurement deficits, and a complex payer environment. Each of these barriers applies with particular force to men’s SP programs. Men are less likely to be aware of SP as an option, less likely to be identified by clinicians as candidates for social referral given the tendency to underreport distress, and less likely to engage with programs that do not fit their masculine self-conception. The measurement deficit is especially acute: existing SP outcome measures have not been validated for populations where help-seeking itself is normatively problematic, and where the therapeutic value of engagement may operate through mechanisms, including behavioural activation, identity affirmation, and peer social connection through shared activity, that standard wellbeing scales do not capture (14). Future SP research should incorporate validated masculinity norms instruments such as the Conformity to Masculine Norms Inventory (CMNI-46) alongside standard outcome measures, to assess whether masculinity-congruent SP programs not only improve wellbeing but shift the normative frameworks that prevent men from accessing support in the first place (15).

The link worker model that Siegel et al. (1) propose as the operational core of SP in the United States requires specific adaptations for male populations. Link workers working with men need training in the dynamics of masculine identity and help-seeking, including understanding that an effective referral to a men’s program may look very different from a referral to a social connection program for other populations. Framing matters: a referral to a woodworking group, a community music program, or a veterans’ activity program will engage men that a referral to a social support group will not, even when the underlying SP rationale is identical. The Canadian Institute for SP’s finding that SP generates a return of $4.43 for every $1 invested (1) likely understates the return for men’s programs specifically, because men’s higher rates of untreated mental illness, substance use, and suicide translate to higher marginal costs of non-engagement and higher marginal benefits of effective connection.

Siegel et al. (1) call for strategic investment in policy frameworks, sustainable financing, workforce development, technology, and research to support system-wide SP adoption. This commentary adds a specific and actionable priority: the development and formal evaluation of masculinity-congruent SP pathways, including Men’s Sheds, veteran music programs, community sports and recreation programs, and other activity-based models with demonstrated male engagement, as distinct and reimbursable SP modalities. The social isolation epidemic that motivates SP is not gender-neutral in its distribution, its causes, or the interventions that can address it. A SP framework that does not explicitly account for how masculine identity shapes men’s relationship to help-seeking will continue to leave untreated the population that bears the greatest burden of undertreated mental illness and the highest cost of non-engagement.
